# Gene Expression Profiling of Grass Carp (*Ctenopharyngodon idellus*) and Crisp Grass Carp

**DOI:** 10.1155/2014/639687

**Published:** 2014-11-30

**Authors:** Ermeng Yu, Jun Xie, Guangjun Wang, Deguang Yu, Wangbao Gong, Zhifei Li, Haiying Wang, Yun Xia, Nan Wei

**Affiliations:** Key Laboratory of Tropical & Subtropical Fishery Resource Application & Cultivation, Ministry of Agriculture, Pearl River Fisheries Research Institute, Chinese Academy of Fishery Sciences, Guangzhou 510380, China

## Abstract

Grass carp (*Ctenopharyngodon idellus*) is one of the most important freshwater fish that is native to China, and crisp grass carp is a kind of high value-added fishes which have higher muscle firmness. To investigate biological functions and possible signal transduction pathways that address muscle firmness increase of crisp grass carp, microarray analysis of 14,900 transcripts was performed. Compared with grass carp, 127 genes were upregulated and 114 genes were downregulated in crisp grass carp. Gene ontology (GO) analysis revealed 30 GOs of differentially expressed genes in crisp grass carp. And strong correlation with muscle firmness increase of crisp grass carp was found for these genes from differentiation of muscle fibers and deposition of ECM, and also glycolysis/gluconeogenesis pathway and calcium metabolism may contribute to muscle firmness increase. In addition, a number of genes with unknown functions may be related to muscle firmness, and these genes are still further explored. Overall, these results had been demonstrated to play important roles in clarifying the molecular mechanism of muscle firmness increase in crisp grass carp.

## 1. Introduction

Freshwater aquaculture plays a very significant role in global aquaculture production. In 2011, 56.8% of the global aquaculture production was freshwater fishes, and output amounted to 35.6 million tons [1]. Grass carp (*Ctenopharyngodon idellus*) is one of the most important freshwater fish that is native to China, and it plays an important role in aquaculture with 4.57 million tons produced in 2011, the highest in fish production worldwide [1]. In China, the grass carp industry aims to increase the production of value-added products in order to improve profitability [2], and crisp grass carp is a kind of high value-added fishes which have firmer muscle and higher contents of crude protein, fat, and amino acids than grass carp [3–5]. Currently in Guangdong province of China, the crisp grass carp has become an economically important freshwater fish because of its increased muscle firmness (hardness).

Fillet firmness of fish is an important quality trait for consumer acceptance in many studies with Chinook and Atlantic salmon [6, 7], channel catfish [8], and gilthead sea bream [9]. Muscle firmness is associated with the intrinsic structure and properties of components of the flesh. It has been found in many studies that firmness is influenced by muscle fiber density, muscle fiber diameter, and intermyofibrillary spaces and gaps [10–13]. These factors are determined by changes in the cellularity of skeletal muscle [14, 15]. The changes in cellularity will contribute to changes in the quality of the skeletal muscle, and since this tissue is the part of the fish destined for human consumption, it may have important economic value [16].

Sole faba bean (*Vicia faba*) feeding differentially enhances muscle firmness of grass carp and the grass carp with higher muscle firmness is called crisp grass carp [17]. Although the composition of faba bean is complex, common characteristic of muscle firmness increase is also demonstrated in other fishes feeding on faba bean including European seabass (*Dicentrarchus labrax*) [18, 19] and channel catfish (*Ictalurus punctatus*) [20]. In the previous studies of crisp grass carp muscle, it has been found that the diameter of muscle fibers was decreased and the content of the ECM was increased [2, 4, 17], and our team also found that the increase in the expression of type I collagen in crisp grass carp is higher than those of grass carp [5]. However, the regulatory mechanism of muscle firmness increase in crisp grass carp is still unclear. Given that the expression of muscle firmness increase was regulated by multiple genes network [21], it is expedient to analyze systematically muscle firmness increase of crisp grass carp in the gene levels using microarray technology, which may help to explore signal transduction pathways of nutritional regulation of fish muscle firmness.

Microarray technology presents a powerful tool for revealing expression patterns and genes associated with phenotypic characteristics [22]. By determination of expression levels of thousands of genes simultaneously in muscle tissue, it could be effective to reveal global gene expression patterns and to identify genes or groups of genes associated with texture variations of Atlantic salmon [21]. In this study, crisp grass carp, having higher muscle firmness, is used to characterize the global gene expression profile in the muscle in comparison with that of grass carp and analyze the biological functions and possible signal transduction pathways that address muscle firmness of crisp grass carp.

## 2. Materials and Methods

### 2.1. Fish

The grass carp and crisp grass carp are raised in six enclosures in the Dongsheng Aquatic Breeding Base (Zhongshan, Guangdong, China), and the diet of grass carp is artificial feed and the diet of crisp grass carp is sole faba bean (*Vicia faba*). The average weights of the specimens were 3.98 ± 0.36 kg (*n* = 60) for crisp grass carp and 3.45 ± 0.52 kg (*n* = 60) for grass carp. In this paper, living fishes were directly dissected and the white muscle tissues of three fishes were obtained for crisp grass carp and grass carp, respectively. The obtained samples were snap-frozen in liquid nitrogen and stored at −80°C for RNA extraction.

### 2.2. RNA Preparation

Total RNA was isolated from white muscle using the TRIzol reagent (Invitrogen, Carlsbad, CA) according to the manufacturer's instruction. The concentration of the isolated RNA was determined by measuring absorbance at 260 nm. The integrity of the RNA was determined by agarose gel electrophoresis and Agilent BioAnalyzer 2100. The RNA was used for microarray analysis and quantitative real-time PCR confirmation.

### 2.3. Microarray, cDNA Labeling, Hybridization, Scanning, and Data Analysis

Affymetrix zebrafish chip containing oligonucleotides representing 14,900 transcripts was used to profile the differences in genes expression of the muscles between crisp grass carp and grass carp. Microarray chips (AFFY-900487) were obtained from Shanghaibio (Shanghai, China). Gene expression levels were determined by comparing the amount of mRNA transcript present in the experimental sample to the control. All experiments were performed following the protocol of Affymetrix Inc. RNA samples of each group were used to generate biotinylated cRNA targets. Hybridizations were performed in the Fluidics Station 450 and chips were scanned using the Affymetrix Scanner 3000. Fluorescent signal intensities for all spots on the arrays were analyzed using the Gene Chip Operating System (GCOS; Affymetrix). Following preprocessing, the data were normalized using global LOWESS normalization. Microarray data were deposited (according to Microarray Gene Expression Data Society Standards) in the NCBI Gene Expression Omnibus (GEO, http://www.ncbi.nlm.nih.gov/geo/) with the series accession number (GSE4787).

### 2.4. GO Category and Pathway Analysis

The categorization of biological process GO (gene ontology) was analyzed using DAVID Bioinformatics Resources 6.7 (http://david.abcc.ncifcrf.gov/). Within the significant category, the enrichment *Re* was given by *Re* = (*n*_*f*_/*n*)/(*N*_*f*_/*N*), where *n*_*f*_ was the number of differential genes within the particular category, *n* was the total number of genes in the same category, *N*_*f*_ was the number of differential genes in the entire microarray, and *N* was the total number of genes in the microarray. The pathway analysis was conducted using KEGG (Kyoto Encyclopedia of Genes and Genomes) database. The false discovery rate (FDR) was calculated to correct the *P* value. *P* value < 0.05 and FDR < 0.05 were used as the threshold to select significant GO categories and KEGG pathways.

### 2.5. Quantitative Real-Time PCR

To validate microarray data, the expression levels of six genes of interest were quantified using real-time PCR with *β*-actin as the internal control. These genes included myostatin (MSTN), collagen type I alpha-1 (COL1A1), collagen type I alpha-2 (COL1A2), and calreticulin (CALR), heat shock cognate 70-kd protein (HSP70), and heat shock protein 90 kDa alpha (HSP90) (Table 1).

The cDNA synthesis was performed using 0.5 *μ*g of DNase-treated total RNA (Turbo DNA-free; Ambion, Austin, TX, USA) using TaqMan Gold Reverse Transcription kit (Applied Biosystems, Foster City, CA, USA) and oligo-dT primers. PCR primers (Table 1) were designed using Vector NTI and synthesized by Invitrogen. The amplicon lengths were checked on 1% agarose gel. PCR efficiency was calculated from tenfold serial dilutions of cDNA for each primer pair in triplicate. Real-time PCR assays were conducted using FastStart SYBR Green Master (Roche Diagnostics, Mannheim, Germany) in an optimized 12 *μ*L reaction volume, using 1 : 10 diluted cDNA, with primer concentrations of 0.4–0.6 *μ*M. PCR was performed in duplicate in 96-well optical plates on Light Cycler 480 (Roche Diagnostics, Mannheim, Germany) under the following conditions: 95°C for 5 min (preincubation), 95°C for 5 s, 60°C for 15 s, 72°C for 15 s (amplification), 95°C for 5 s, and 65°C for 1 min (melting curve). 45 cycles were performed. Relative expression of mRNA was evaluated using the ΔΔCT method. Statistical differences were determined by one-way ANOVA followed by Duncan's multiple range test (*P* < 0.05). All statistics were performed using software SPSS 15.0.

## 3. Results

**Figure 1:**
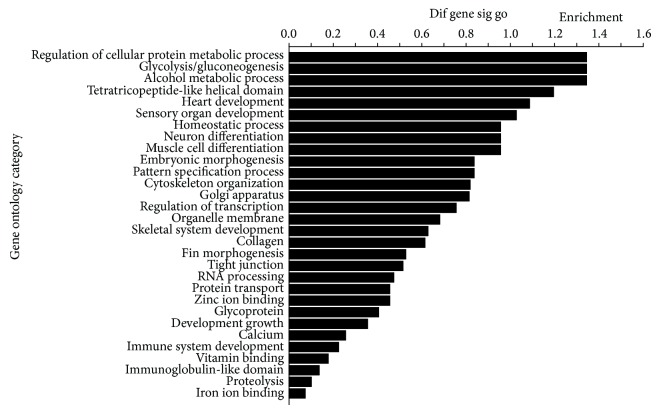
GO category based on biological process for differentially expressed genes. Vertical axis was the GO category and horizontal axis was the enrichment of GO.

The microarray analysis demonstrated that expressions of 127 genes were upregulated and 114 genes were downregulated in the muscle of crisp grass carp compared with the control group. According to the genes of differential expression, the biological processes GO terms mainly consisted of protein metabolism, muscle development and growth, carbohydrate metabolism, and so on (Figure 1).

### 3.1. Genes Involved in Protein Metabolism

Differentially expressed genes involved in protein metabolism in the muscle of crisp grass carp and grass carp were shown in Table 2. Expressions of collagen type I alpha-1 and alpha-2, type II alpha-1a were upregulated in the muscle of crisp grass carp. Differentially expressed genes involved in the protein metabolism were clustered into biological categories including protein transport (9 genes), proteolysis (9 genes), and regulation of cellular protein metabolic process (4 genes). The 11 genes that regulate the glycoproteins were found with nine notably upregulated and two downregulated.

### 3.2. Genes Involved in Muscle Development and Growth

The genes involved in muscle development and growth were classified into developmental growth (4 genes), muscle cell differentiation (4 genes), skeletal system development (4 genes), and cytoskeleton organization (14 genes) in the crisp grass carp. Above all, transcription of MSTN, which was tightly related to muscle development, was upregulated in the muscle of crisp grass carp (Table 3). In addition, the mRNAs of three genes responsible for tight junction were upregulated.

### 3.3. Genes Involved in Carbohydrate Metabolism

Downregulated expressions of glycolytic enzymes were detected in the muscle of crisp grass carp (Table 4). These enzymes include enolase-3, hexokinase-1, hexokinase-2, phosphofructokinase, pyruvate dehydrogenase, glycerophosphodiester phosphodiesterase and phosphatase, and tensin homolog-B.

### 3.4. Genes Involved in Calcium and Other Ions' Metabolism

In the muscle of crisp grass carp, fifty-five differentially expressed genes related to metal ions were detected. The GOs of these genes included zinc ion binding, calcium and iron ion binding (Table 5). As genes involved in vitamin metabolism, cysteine conjugate-beta lyase and KATIII were upregulated.

### 3.5. Genes Involved in Nucleic Acid Metabolism

The differential expression of genes involved in protein biosynthesis occurred at multiple levels, including regulation of transcription (31 genes), RNA processing (6 genes), and tetratricopeptide-like helical domain (8 genes) (Table 6).

### 3.6. Genes of Differential Expression Involved in Other GOs

Fifty-two differentially expressed genes were included in the GO terms of immune system development and immunoglobulin-like domain (13 genes), embryonic morphogenesis (9 genes), Golgi apparatus (6 genes), neuron differentiation (8 genes), organelle membrane (13 genes), and fin morphogenesis (3 genes) (Table 7).

### 3.7. Pathway Analysis

**Figure 2:**
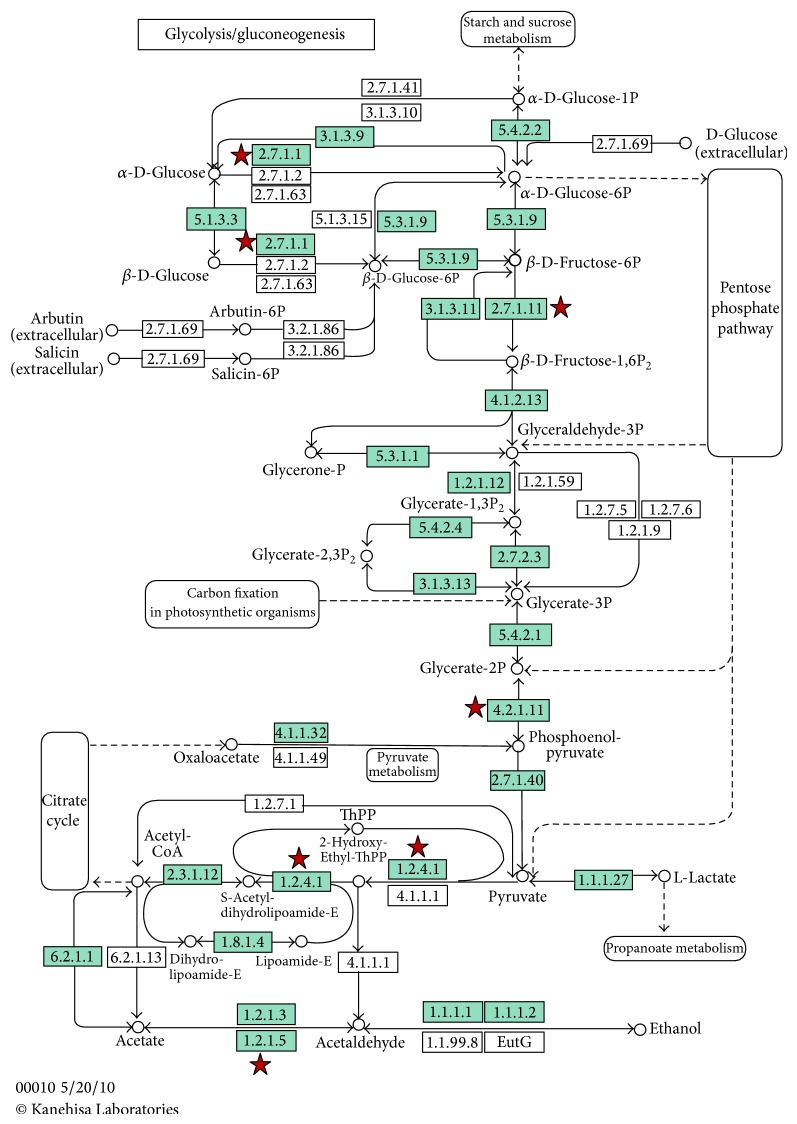
Pathway information of glycolysis/gluconeogenesis. Green boxes denote signaling pathway protein. Red stars mark the genes of differential expression including upregulated and downregulated genes: box 2.7.1.1 for hexokinase-1 and hexokinase-2, box 2.7.1.11 for phosphofructokinase (muscle a), box 4.2.1.11 for enolase-3 (beta, muscle), box 1.2.4.1 for pyruvate dehydrogenase (lipoamide) alpha-1a, and box 1.2.1.5 for aldehyde dehydrogenase-3 family (member D1). Figure 2 was formed from all differentially expressed genes that were analysed using DAVID Bioinformatics Resources 6.7 (http://david.abcc.ncifcrf.gov/).

To further analyze the interactional relation of all differentially expressed genes, the KEGG pathway analysis was used in this study. The results of pathway analysis found that downregulated signals in glycolysis/gluconeogenesis pathway happened in the crisp grass carp (*P* value < 0.01). The detailed information of glycolysis/gluconeogenesis pathway was shown in Figure 2, which was formed from all differentially expressed genes.

### 3.8. Quantitative Real-Time PCR

**Figure 3:**
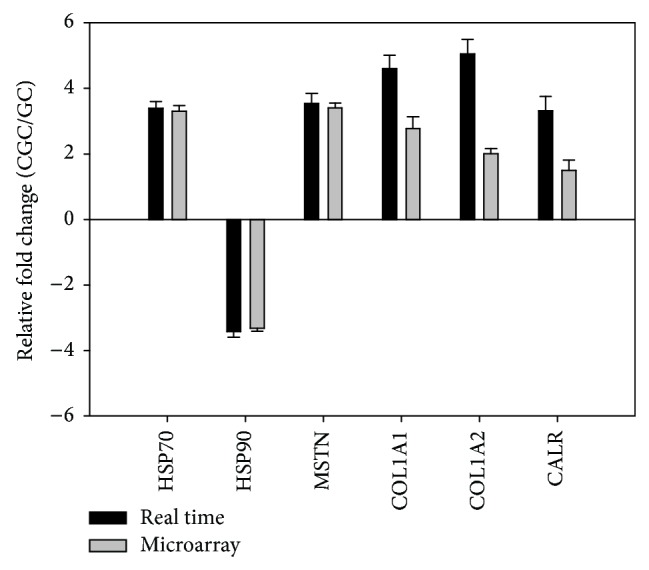
Quantitative real-time PCR confirmation of six differentially expressed genes identified by microarray analysis in crisp grass carp (CGC) versus grass carp (GC). Three samples were used for quantitative real-time PCR confirmation for experimental group and control group. *β*-Actin gene was used as an internal control. HSP70 for heat shock cognate 70-kd protein, HSP90 for heat shock protein 90 kDa alpha (cytosolic, B1), MSTN for myostatin, COL1A1 for type I collagen (alpha-1), COL1A2 for type I collagen (alpha-2), and CALR for calreticulin. Differential expression was determined by one-way ANOVA (*P* < 0.05).

To verify the data obtained by microarray analysis, quantitative real-time PCR was performed for six genes, including five upregulated genes and one downregulated gene, with a *β*-actin gene used as an internal control. The relative hybridization intensities of the six selected genes are basically consistent with those analyzed by real-time PCR, thus confirming that use of the zebrafish genome array was suitable for this study (Figure 3).

## 4. Discussion

In this study, we show that muscle firmness increase of crisp grass carp is tightly related to the genes of differential expression in the functional groups including differentiation of muscle fibers, deposition of extracellular matrix (ECM), glycolysis/gluconeogenesis pathway, and calcium metabolism.

### 4.1. Genes Involved in Differentiation of Muscle Fibers

The decrease in the diameter of muscle fibers in crisp grass carp may be related to the downregulated expressions of MSTN and axin and differentially expressed genes involved in diminution of actin filaments. MSTN, known as growth differentiation factor (GDF)-8, was reported to inhibit the proliferation and differentiation of resident muscle cell [23]. In this study, evidence that the growth of muscle cell is inhibited in the muscle of crisp grass carp is that, in the muscle of crisp grass carp, the transcription level of MSTN is elevated from microarray expression, and the mRNA expression of MSTN is 3.4 times that of grass carp by gene quantitative analysis. It was found that the muscle fibres of crisp grass carp were less than those of grass carp [17]. As it is an important protein constituting muscle, diminution of actin filaments in crisp grass carp muscle has been suggested by the differential expressions of genes related to actin. The downregulation of actin related protein 2/3 complex and nexilin and upregulation of capping protein in this paper suggested the diminution of the actin filaments in crisp grass carp muscle [24, 25].

### 4.2. Genes Involved in Deposition of ECM

In our experiments, muscle firmness increase of the crisp grass carp has been demonstrated in the increasing deposition of ECM, which includes upregulated expressions of collagen and differential expressions of transforming growth factor-*β*1 (TGF-*β*1), and the genes related to fibroblasts. As an important protein of ECM, collagen has been proven to be closely related to the firmness of muscle in fish [26, 27]. In zebrafish, deposits of collagen were positively related to the increase in muscle firmness [27]. It was documented that downregulated expression of collagen and collagenase-3 enzyme elicited reduction in the firmness of atrophying muscle of rainbow trout [26]. Our results that the expressions of type I alpha-1 and alpha-2 and type II alpha-1a collagen are upregulated in crisp grass carp are consistent with the fact that collagen content in the muscle of crisp grass carp was 1.36 times greater than that of grass carp [17]. And the increase in the collagen content plays an important role in firmness increase of crisp grass carp muscle and resultant texture characteristics [2, 4]. For the upregulated expressions of type I and type II collagen, we speculate that the genes of INF-7 and procollagen-proline 4-hydroxylase (an enzyme hydrolyzing the collagen) probably play important roles in crisp grass carp. The basal expression of type I collagen was inhibited by INF-7 acting in not definitively located promoter region [28]. In the muscle of crisp grass carp, the mRNA level of INF-7 was 0.18 times that of grass carp and the transcription levels of type I and type II collagen increased. The results suggest that the inhibition of expression of INF-7 in the muscle of crisp grass carp promotes the synthesis of type I and type II collagen. The expression of procollagen-proline 4-hydroxylase was downregulated in the muscle of crisp grass carp, and this may help understand the increase in the expression of type I and type II collagen.

The increasing deposition of ECM in crisp grass carp muscle can also be suggested by enhanced TGF-*β*1 signaling. Enhanced TGF-*β*1 signaling in crisp grass carp could be demonstrated in the differential expressions including upregulated expression of TGF*β*-induced factor homeobox-1 and downregulated expressions of both interferon regulatory factor-7 and interferon-inducible protein-kinase which are closely related to interferon inhibition of TGF-*β*1 signaling pathway. TGF-*β*1 is a pleiotropic cytokine known to play an important role in cell growth, embryonic development, and tissue repair and could induce the synthesis and accumulation of components of the extracellular matrix (ECM) in the muscle [29]. It was found that upregulated expression of TGF-*β*1 correlated with increase of ECM in the muscle [30]. In addition, the significantly upregulated expression of activin A receptor in crisp grass carp in this study, a downstream gene of TGF-*β*1 signaling pathway, further confirms enhanced TGF-*β*1 signaling.

Besides TGF-*β*1, the genes related to fibroblasts may play important roles in the increasing deposition of ECM. Fibroblasts were proven to produce an accumulation of fibrotic interstitial ECM components such as collagen and fibronectin [31], growth factors [32], and cytokines [33]. In the muscle of crisp grass carp, the mRNA level of fibroblast growth factor receptor 4 was more than that of grass carp, suggesting that fibroblast was activated. Some evidences had shown that MSTN could directly stimulate muscle fibroblast proliferation [34], and the enhancement in the transcripts levels of MSTN further demonstrated fibroblast proliferation in the muscle of crisp grass carp. Stromal cell-derived factor (SDF) was also involved in the activation, proliferation, and migration of fibroblast and secretion of ECM [35] and played important roles in fibrosis [36]. Both SDF-1 and SDF-4 had been demonstrated to be highly expressed in the muscle of crisp grass carp than those in grass carp, suggesting that these two genes may be responsible for the increased firmness in the muscle of crisp grass carp.

### 4.3. Genes Involved in Glycolysis/Gluconeogenesis Pathway

Downregulated expressions of genes involved in glycolysis, as a major source of energy in the muscle, could result in the lower expression of myofiber proteins which were closely related to muscle firmness increase [21]. In this study, downregulated glycolysis/gluconeogenesis pathway in the crisp grass carp may contribute to the muscle firmness increase of crisp grass carp. The evidence that glycolysis pathway in the muscle of crisp grass carp is downregulated is the decrease in the expressions of five glycolytic enzymes in addition to aldehyde dehydrogenase which acts on products of glycolysis. The evidence that crisp grass carp have lower levels of anaerobic metabolism is that they have lower velocity of both glycolysis and TCA cycle. On the contrary, higher rates of aerobic metabolism in crisp grass carp are demonstrated by the upregulated expressions of mitochondrial genes. Larsson et al. also found that the firmness of Atlantic salmon muscle was associated with high rates of aerobic metabolism [21]. In addition, differential expression of genes involved in glucose utilization has also been found in the change of muscle texture under nutrition restriction [26, 37, 38]. However, the function and mechanism of glucose utilization acting in the muscle firmness increase still need further exploration.

### 4.4. Genes Involved in Calcium Metabolism

Calcium could activate the increase in the density of filamentous myosins [39], and the increasing density of filamentous myosins contributes to the muscle firmness increase [10]. The previous results that the calcium content in crisp grass carp [40] and the density of filamentous myosins were increased [17] could further help to understand the muscle firmness increase in crisp grass carp. In this paper, the expressions of seven genes related to calcium including calreticulin (CRT), calmodulin (CaM), and cadherin protein (Cad) were found with upregulated mRNA expression. CRT, as one of the major calcium-binding proteins of the endoplasmic reticulum, was involved in the regulation of intracellular Ca^2+^ homeostasis and endoplasmic reticulum Ca^2+^ storage capacity [41], and its overexpression increased calcium fluxes across endoplasmic reticulum [42]. CaM, a protein that binds calcium with high affinity and specificity, serves as an intracellular Ca^2+^-receptor and mediates the Ca^2+^ regulation of cyclic nucleotide and glycogen metabolism, secretion, motility, and Ca^2+^ transport [43]. Thus, an increase in the Ca^2+^ content of crisp grass carp muscle [40] further contributed to increased expression levels of calcium-dependent proteins including CaM and Cad [44]. In addition, downregulated expressions of three genes including desmocollin, guanylate cyclase activator, and zgc:136759 would help the study of calcium regulation in crisp grass carp.

### 4.5. Heterohybridization to Zebrafish cDNA Microarray and Its Application to Grass Carp

Affymetrix zebrafish chip has been proven to be a valid way to examine the gene expression profiling of grass carp muscle. Because DNA microarrays are unavailable for grass carp, the Affymetrix zebrafish chip was used in this study. Grass carp is near to the zebrafish in an evolutionary sense, and these two species were a family of Cyprinidae. The Affymetrix zebrafish array had been used to screen gene transcript profiles of grass carp recently, and the 416 genes of differential expressions were found to be related to the use of LS as an alternative dietary antibiotic in fish [45]. Use of cross-hybridization with microarrays for analysis of closely related species also had been reported by other researchers. A cDNA microarray from African cichlid fish,* Astatotilapia burtoni*, had been proven to be a powerful tool for analyzing the transcription profile of other cichlid species including* Enantiopus melanogenys* and* Neolamprologus brichardi* and* Oreochromis niloticus* [46]. The microarray composed of channel catfish (*Ictalurus punctatus*) transcripts was effectively used to analyze gene expression profiling of blue catfish (*Ictalurus furcatus*) [47]. The Affymetrix zebrafish array was also used to screen gene expression profiles of distantly related species, and it was found that 375 genes were significantly expressed in the muscle tissues of Chinese mandarin fish (*Siniperca chuatsi*) [48]. Such applications indicated that use of the zebrafish genome array could be a valid way to examine grass carp, and a conclusion was strongly supported in the current study by real-time RT-PCR validation.

In conclusion, during the muscle firmness increase from grass carp to crisp grass carp, a total of 127 transcripts were found to be upregulated and a total of 114 transcripts were downregulated. Strong correlation with muscle firmness increase of crisp grass carp was found for these genes from differentiation of muscle fibers and deposition of ECM, and also glycolysis pathway and calcium metabolism may contribute to muscle firmness increase. However, a number of genes with unknown functions may be related to muscle firmness, and these genes can be regarded as candidate markers of nutritional regulation of grass carp muscle firmness.

## Figures and Tables

**Table 1:** Primers used in quantitative real-time PCR.

Gene name	Forward primer (5′→3′)	Reverse primer (5′→3′)	GenBank accession number
HSP70	GTGTGAGCGAGCCAAGAGAA	TTGTTGATCCACCAACCAGAA	FJ483832
HSP90	GCCGTGGAACCAGAGTCATT	ATCTCCTTGTCGCGTTCCTT	FJ517554
MSTN	TGCCACCACAGAGACCATCA	TGTGTCTTCCTCCGTCCGTAA	EU555520
COL1A1	GCATGGGGCAAGACAGTCA	ACGCACACAAACAATCTCAAGT	HM363526.1
COL1A2	ACATTGGTGGCGCAGATCA	ACTCCGATAGAGCCCAGCTT	HM771241.1
CALR	AGGCAGAACCACCTAATCAA	CCACCTTCTCGTTGTCGATTT	HQ444741.1
*β*-Actin	TGACGAGGCTCAGAGCAAGA	GCAACACGCAGCTCGTTGTA	M25013

**Table 2:** Differentially expressed genes involved in protein metabolism in the muscle of crisp grass carp and grass carp.

Gene name	Affy-ID	Fold change
Collagen		
Collagen, type I, alpha-2	DrAffx.2.1.S1_s_at	5.049230
Collagen, type I, alpha-1	Dr.1377.1.A1_at	4.646780
Collagen type II, alpha-1a	Dr.3761.1.S1_at	4.283097
si:ch211-106n13.3	Dr.1276.1.A1_at	1.825742
Procollagen-proline, 2-oxoglutarate 4-dioxygenase (proline 4-hydroxylase), alpha polypeptide-2; hypothetical protein LOC100151456	Dr.19144.1.A1_at	0.253430
C1q and tumor necrosis factor related protein-5	Dr.965.1.S1_at	0.212245
Proteolysis		
Ubiquitin specific protease-16	Dr.21873.1.A1_at	4.844206
Phosphate regulating gene with homologues to endopeptidases on the X chromosome	Dr.25529.1.S1_at	1.544958
Ubiquitin-conjugating enzyme E2E 3 (UBC4/5 homolog, yeast)	Dr.23793.1.A1_at	0.581777
zgc:123295	Dr.18631.1.S1_at	0.502692
zgc:92791; hypothetical LOC797742	Dr.15777.1.A1_at	0.396054
Carboxypeptidase-A5	Dr.4882.1.S1_at	0.333607
Speckle-type POZ protein	Dr.12893.1.S1_at	0.202174
Six-cysteine containing astacin protease 1	Dr.21939.1.A1_at	0.201674
Janus kinase-1	Dr.18349.1.A1_at	0.188976
Protein transport		
zgc:92303	Dr.4306.1.A1_at	12.18947
zgc:77724	Dr.14413.1.A1_at	4.213999
KDEL (Lys-Asp-Glu-Leu) endoplasmic reticulum protein retention receptor 2, like	Dr.1198.1.A1_at	2.799246
Clathrin, light chain (Lca)	Dr.26380.1.A1_at	1.98067
Translocase of inner mitochondrial membrane 17 homolog A (yeast)	Dr.3096.1.A1_at	1.92503
Importin-7	Dr.19552.1.S1_at	0.523015
Adaptor-related protein complex 1, sigma 1 subunit	Dr.18735.1.A1_at	0.409458
Similar to peroxisome biogenesis factor 13; peroxisome biogenesis factor 13	Dr.6902.1.S1_at	0.321697
Chromatin modifying protein 4B	Dr.16859.1.S1_at	0.274372
Regulation of cellular protein metabolic process		
Eukaryotic translation initiation factor-5A	Dr.20010.3.S2_at	3.296618
Neuroguidin, EIF4E binding protein	Dr.20137.1.S1_at	2.291049
Axin 1	Dr.17733.1.S1_at	0.505078
MAP kinase-interacting serine/threonine kinase 2b	Dr.17570.1.S3_at	0.449659
Glycoprotein		
Rhesus blood group, B glycoprotein	Dr.9532.1.S1_at	8.115116
Stromal cell-derived factor-4	Dr.20092.1.S1_at	4.935459
Acetylcholinesterase	Dr.15722.1.S1_at	4.445239
Melatonin receptor type 1B a	Dr.20978.1.S1_at	3.767983
Semaphorin 3ab	Dr.8112.1.S1_at	3.37293
Myelocytomatosis oncogene b	Dr.16048.1.S1_at	3.10492
zgc:123242	Dr.13408.1.S1_at	2.546485
Ephrin-A2	Dr.20957.2.A1_at	1.785163
Fibroblast growth factor receptor-4	Dr.409.1.S1_at	1.746774
Opsin 1 (cone pigments), long-wave-sensitive, 1	Dr.131175-1_s_at	0.379971
Transmembrane protein-192	Dr.17388.2.S1_at	0.214102

Fold change = (signal intensity of a gene in the muscle of crisp grass carp)/(signal intensity of the gene in the muscle of grass carp).

**Table 3:** Differentially expressed genes involved in muscle development and growth in the muscle of crisp grass carp and grass carp.

Gene name	Affy-ID	Fold change
Developmental growth		
Chemokine (C-X-C motif) ligand 12a (stromal cell-derived factor-1)	Dr.822.1.S3_at	3.862025
Myostatin (MSTN)	Dr.5778.1.S1_at	3.411231
Axin 1	Dr.17733.1.S1_at	0.505078
Survival of motor neuron protein interacting protein-1	Dr.2724.1.S1_at	0.480291
Muscle cell differentiation		
Acetylcholinesterase	Dr.15722.1.S1_at	4.445239
Chemokine (C-X-C motif) ligand 12a (stromal cell-derived factor 1)	Dr.822.1.S3_at	3.862025
Glycogen synthase kinase 3-alpha	Dr.259.1.S1_at	0.663178
Pre-B-cell leukemia transcription factor-1a; zgc:1588-24; pre-B-cell leukemia transcription factor-4; hypothetical LOC100004634; hypothetical protein LOC100150879	Dr.4926.1.S1_at	0.175405
Cytoskeleton organization		
Acetylcholinesterase	Dr.15722.1.S1_at	4.445239
Chemokine (C-X-C motif) ligand 12a (stromal cell-derived factor 1)	Dr.822.1.S3_at	3.862025
zgc:158673	Dr.4838.1.A1_at	2.108265
Capping protein (actin filament) muscle Z-line, beta	Dr.25474.1.S1_at	1.611818
Tubulin, beta-2c	Dr.5605.3.S1_x_at	0.566588
Actin related protein 2/3 complex, subunit 4, like; actin related protein 2/3 complex, subunit 4	Dr.5314.1.S1_at	0.459813
Septin-8a	Dr.4204.1.A1_at	0.388101
Similar to RP11-100C15.2	Dr.21663.1.A1_at	0.284452
Lamin-B1	Dr.25051.1.S2_at	0.249782
ADP-ribosylation factor-like 8-Ba	Dr.7615.1.A1_at	0.249671
zgc:136930	Dr.24487.1.A1_at	0.233076
Hypothetical protein LOC553488	Dr.26067.1.A1_s_at	0.221494
Janus kinase-1	Dr.18349.1.A1_at	0.188970
Nexilin (F actin binding protein)	Dr.4859.1.A1_at	0.139300
Skeletal system development		
Activin A receptor, type I like	Dr.606.1.S2_at	11.01876
Eukaryotic translation initiation factor-3, subunit E, a/b	Dr.5119.1.A1_at	4.241705
Cytochrome P-450, family-26, subfamily b, polypeptide 1	Dr.180.1.A1_at	3.092533
Runt-related transcription factor-3	Dr.10668.1.S2_at	0.174100
Tight junction		
zgc:110333; zgc:173444	Dr.10400.1.A1_at	3.965793
Tight junction protein-3	Dr.21038.1.S1_at	3.436695
Occludin-alpha	Dr.7692.1.A1_at	2.112512

Fold change = (signal intensity of a gene in the muscle of crisp grass carp)/(signal intensity of the gene in the muscle of grass carp).

**Table 4:** Differentially expressed genes involved in carbohydrate metabolism in the muscle of crisp grass carp and grass carp.

Gene name	Affy-ID	Fold change
Glycolysis/gluconeogenesis		
Aldehyde dehydrogenase-3 family, member D1	Dr.4094.1.S1_at	5.510521
T-box 24	Dr.18309.1.S1_at	5.279783
zgc:55970	Dr.24685.1.S1_at	1.887322
Hexokinase-2	Dr.10553.1.S1_at	1.760913
Enolase-3 (beta, muscle)	Dr.9746.4.S1_at	0.640655
Phosphofructokinase, muscle a	Dr.13621.1.A1_at	0.615103
Cytochrome c oxidase, subunit VIIc	Dr.7444.1.S1_at	0.380282
Cytochrome c oxidase subunit 1	Dr.20553.2.A1_at	0.223735
Pyruvate dehydrogenase (lipoamide) alpha-1a	Dr.2656.1.A1_at	0.152896
hexokinase-1	Dr.25364.1.A1_s_at	0.121231
Alcohol metabolic process		
Acetylcholinesterase	Dr.15722.1.S1_at	4.445239
Hexokinase-2	Dr.10553.1.S1_at	1.760913
Phosphofructokinase, muscle a	Dr.13621.1.A1_at	0.645103
Enolase-3 (beta, muscle)	Dr.9746.4.S1_at	0.600655
Glycerophosphodiester phosphodiesterase domain containing-3	Dr.10416.1.S1_at	0.274577
Phosphatase and tensin homolog B (mutated in multiple advanced cancers 1)	Dr.5559.3.A1_at	0.195537
Pyruvate dehydrogenase (lipoamide) alpha 1a	Dr.2656.1.A1_at	0.152896
Hexokinase-1	Dr.25364.1.A1_s_at	0.121231

Fold change = (signal intensity of a gene in the muscle of crisp grass carp)/(signal intensity of the gene in the muscle of grass carp).

**Table 5:** Differentially expressed genes involved in metal ions and vitamin metabolism in the muscle of crisp grass carp and grass carp.

Gene name	Affy-ID	Fold change
Calcium		
Rhomboid, veinlet-like 3 (*Drosophila*); hypothetical LOC100005244	Dr.25770.2.A1_at	6.680544
Stromal cell-derived factor-4	Dr.20092.1.S1_at	4.935459
Protocadherin-10a	Dr.21790.1.A1_at	4.905736
Calreticulin	Dr.25177.1.S1_at	3.450125
zgc:123242	Dr.13408.1.S1_at	2.546485
Parvalbumin-3	Dr.15359.1.S1_at	1.997458
Calmodulin 2b, /// calmodulin 3b /// calmodulin 3a /// calmodulin 2a /// calmodulin 1b /// zgc:55813 /// calmodulin 1a	Dr.7638.1.S1_at	1.549420
zgc:136759	Dr.19975.1.S1_at	0.336455
Guanylate cyclase activator 1-A	Dr.12592.1.S1_at	0.30074
Desmocollin 2-like	Dr.934.1.A1_at	0.23301
Iron ion binding		
zgc:92245; hypothetical LOC792323	Dr.20662.1.A1_at	4.470659
Transferrin-a; Rho-class glutathione S-transferase	Dr.1889.1.S1_at	3.685072
Cytochrome P450, family 26, subfamily b, polypeptide-1	Dr.180.1.A1_at	3.092533
Cytochrome c oxidase, subunit VIIc	Dr.7444.1.S1_at	2.380282
Cytochrome c oxidase subunit 1	Dr.20553.2.A1_at	2.223735
Procollagen-proline, 2-oxoglutarate 4-dioxygenase (proline 4-hydroxylase), alpha polypeptide 2; hypothetical protein LOC100151456	Dr.19144.1.A1_at	0.25343
Zinc ion binding		
Similar to zinc finger protein-135 (zinc finger protein 61) (zinc finger protein 78-like 1); zgc:174288; zgc:110249	Dr.11066.2.A1_a_at	13.94189
si:ch211-222k6.1; si:ch211-222k6.2; zgc:174564	Dr.23520.1.A1_at	12.04316
Similar to COASTER	Dr.14346.2.A1_x_at	11.16127
zgc:174263	Dr.15456.1.A1_at	6.072371
Muscleblind-like (*Drosophila*); hypothetical protein LOC100150760; hypothetical protein LOC100150761	Dr.25973.1.A1_at	6.004707
Ubiquitin specific protease-16	Dr.21873.1.A1_at	4.844206
si:rp71-15k1.2; myeloid/lymphoid or mixed-lineage leukemia-4a	Dr.9377.1.A1_at	4.679487
si:ch211-45m15.2	Dr.8200.1.S1_at	4.653204
zic family member-2 (odd-paired homolog, *Drosophila*) b	Dr.10614.1.A1_at	4.526444
zgc:77724	Dr.14413.1.A1_at	4.213999
zgc:162730	Dr.3936.1.A1_at	3.742291
RNA binding motif protein 4.1	Dr.21594.1.A1_at	3.487187
l(3)mbt-like 2 (*Drosophila*)	Dr.17271.1.A1_at	3.197401
LIM and SH3 protein-1	Dr.25320.1.A1_at	2.882738
LIM and calponin homology domains-1	Dr.10986.1.A1_at	2.689072
zgc:158673	Dr.4838.1.A1_at	2.108265
si:ch73-38p6.1	Dr.3142.1.S1_at	1.823564
zgc:56116	Dr.745.1.A1_at	1.590587
Phosphate regulating gene with homologues to endopeptidases on the X chromosome	Dr.25529.1.S1_at	1.544958
Zinc finger protein-207, a	Dr.93.1.A1_a_at	0.65725
Retinoic acid receptor, alpha b	Dr.305.1.S1_at	0.566773
Alpha thalassemia/mental retardation syndrome X-linked, like; alpha thalassemia/mental retardation syndrome X-linked homolog (human)	Dr.26404.2.S1_at	0.502432
APC11 anaphase promoting complex subunit 11 homolog	Dr.18189.1.S1_at	0.491208
zgc:174649; similar to zinc finger protein-180 (HHZ168); hypothetical LOC570013; zgc:174651; similar to zinc finger protein-560; zgc:173603	Dr.21894.1.A1_at	0.474262
zgc:56628	Dr.18443.1.S1_at	0.462863
Carboxypeptidase-A5	Dr.4882.1.S1_at	0.333607
zgc:153061	Dr.6513.1.A1_a_at	0.323466
zgc:153171; F-box only protein 11-like	Dr.12361.1.S1_at	0.308205
wu:fi20g04	Dr.7293.1.S1_at	0.262579
Novel protein similar to human rearranged L-myc fusion sequence (RLF)	Dr.15042.1.A1_at	0.256942
si:dkeyp-89d7.1	Dr.4953.1.S1_at	0.236327
Zinc finger, FYVE domain containing 21	Dr.3023.1.S1_at	0.216677
wu:fd12d03	Dr.2692.1.A1_at	0.210638
Six-cysteine containing astacin protease-1	Dr.21939.1.A1_at	0.201674
Janus kinase-1	Dr.18349.1.A1_at	0.188976
zgc:158455	Dr.15141.1.A1_at	0.184993
LIM homeobox-1a	Dr.24443.1.A1_at	0.182767
Zinc fingers and homeoboxes 3	Dr.26180.1.A1_at	0.150721
Transcription elongation factor A (SII), 3	Dr.15634.1.S1_at	0.093517
Vitamin binding		
Cysteine conjugate-beta lyase; cytoplasmic (glutamine transaminase K, kynurenine aminotransferase)	Dr.2828.2.A1_at	9.133776
Similar to Kynurenine-oxoglutarate transaminase-3 (Kynurenine-oxoglutarate transaminase III) (Kynurenine aminotransferase III) (KATIII) (cysteine-S-conjugate beta-lyase 2); cysteine conjugate-beta lyase-2	Dr.18800.1.S1_at	1.518084
Procollagen-proline, 2-oxoglutarate 4-dioxygenase, alpha polypeptide 2; hypothetical protein LOC100151456	Dr.19144.1.A1_at	0.25343

Fold change = (signal intensity of a gene in the muscle of crisp grass carp)/(signal intensity of the gene in the muscle of grass carp).

**Table 6:** Differentially expressed genes involved in nucleic acid metabolism in the muscle of crisp grass carp and grass carp.

Gene name	Affy-ID	Fold change
Regulation of transcription		
T-box 24	Dr.18309.1.S1_at	5.279783
Ubiquitin specific protease-16	Dr.21873.1.A1_at	4.844206
si:ch211-45m15.2	Dr.8200.1.S1_at	4.653204
Neurogenin-1	Dr.555.1.S1_at	4.347208
zgc:152921	Dr.21248.1.A1_at	3.604867
l(3)mbt-like 2 (*Drosophila*)	Dr.17271.1.A1_at	3.197401
Myelocytomatosis oncogene b	Dr.16048.1.S1_at	3.10492
zgc:92106	Dr.7710.1.A1_at	2.86037
E74-like factor 2 (ets domain transcription factor)	Dr.2328.1.S1_at	2.775788
zgc:153012	Dr.17420.1.S1_at	2.482655
TGFB-induced factor homeobox-1	Dr.139.1.S1_at	2.45607
Ventral anterior homeobox-1	DrAffx.1.61.S1_at	2.194558
Diencephalon/mesencephalon homeobox-1a	Dr.18845.1.S1_at	2.072013
Homeobox B4a	Dr.15716.1.S1_at	2.071785
CCR4-NOT transcription complex, subunit 3b	Dr.6354.2.A1_x_at	1.938399
Telomeric repeat binding factor (NIMA-interacting) 1	Dr.25530.1.A1_at	0.662482
Retinoic acid receptor, alpha b	Dr.305.1.S1_at	0.566773
Activating transcription factor-7 interacting protein	Dr.433.2.S1_at	0.312221
zgc:162976	Dr.6862.1.A1_at	0.278785
si:dkey-211g8.3	Dr.18812.1.S1_at	0.256496
Sine oculis-related homeobox-6a	Dr.26486.1.S1_at	0.243693
Suppressor of Ty 6 homolog (*S. cerevisiae*)	Dr.6422.1.S1_at	0.240522
POU domain gene-12	Dr.37.2.S1_at	0.230685
wu:fd12d03	Dr.2692.1.A1_at	0.210638
Heat shock transcription factor-1	Dr.8301.1.S1_a_at	0.193731
Interferon regulatory factor-7	Dr.10428.1.S1_at	0.184330
LIM homeobox-1a	Dr.24443.1.A1_at	0.182767
Pre-B-cell leukemia transcription factor 1a; zgc:158824; pre-B-cell leukemia transcription factor 4; hypothetical LOC100004634; hypothetical protein LOC100150879	Dr.4926.1.S1_at	0.175405
Runt-related transcription factor-3	Dr.10668.1.S2_at	0.174100
Zinc fingers and homeoboxes-3	Dr.26180.1.A1_at	0.150721
Transcription elongation factor A (SII), 3	Dr.15634.1.S1_at	0.093517
RNA processing		
Trinucleotide repeat containing-4	Dr.8323.1.S1_at	6.653872
Molybdenum cofactor synthesis-3	Dr.13391.1.S1_at	2.424007
PRP39 pre-mRNA processing factor 39 homolog (yeast)	Dr.340.1.S1_at	1.852201
Survival of motor neuron protein interacting protein-1	Dr.2724.1.S1_at	0.480291
XPA binding protein-2	Dr.12501.1.A1_at	0.348226
Polypyrimidine tract binding protein-1a	Dr.20803.2.S1_at	0.166418
Tetratricopeptide-like helical domain		
Tetratricopeptide repeat domain-8	Dr.11994.1.A1_at	2.342792
PRP39 pre-mRNA processing factor 39 homolog (yeast)	Dr.340.1.S1_at	1.852201
Tetratricopeptide repeat domain-5	Dr.6679.1.A1_at	0.463885
Sperm associated antigen-1	Dr.9954.1.A1_at	0.355814
XPA binding protein-2	Dr.12501.1.A1_at	0.348226
FIC domain containing	Dr.13784.1.A1_at	0.337339
Procollagen-proline, 2-oxoglutarate 4-dioxygenase (proline 4-hydroxylase), alpha polypeptide 2; hypothetical protein LOC100151456	Dr.19144.1.A1_at	0.253430
Protein-kinase, interferon-inducible double stranded RNA dependent inhibitor	Dr.10669.1.S1_at	0.182015

Fold change = (signal intensity of a gene in the muscle of crisp grass carp)/(signal intensity of the gene in the muscle of grass carp).

**Table 7:** Differentially expressed genes involved in other GOs (gene ontology) in the muscle of crisp grass carp and grass carp.

Gene name	Affy-ID	Fold change
Immune system development		
Activin A receptor, type I like	Dr.606.1.S2_at	11.01876
Heat shock cognate 70-kd protein	NM_131397.2	3.284166
SNF related kinase-1	Dr.25951.1.A1_at	3.016243
Heat shock protein 90 kDa alpha, cytosolic, B1	NM_131310.1	0.302405
runt-related transcription factor-3	Dr.10668.1.S2_at	0.174100
Immunoglobulin-like domain		
Major histocompatibility complex class I UDA gene	Dr.22347.1.S1_at	11.02728
Neural cell adhesion molecule-2	Dr.12598.1.S1_at	5.365372
Semaphorin-3ab	Dr.8112.1.S1_at	3.37293
Fibroblast growth factor receptor-4	Dr.409.1.S1_at	1.746774
Junctional adhesion molecule-3	Dr.4725.1.A1_at	0.618889
Basigin	Dr.16700.1.A1_at	0.305291
Nexilin (F actin binding protein)	Dr.4859.1.A1_at	0.139300
zgc:171897	Dr.21314.1.A1_at	0.116185
Embryonic morphogenesis		
Activin A receptor, type I like	Dr.606.1.S2_at	11.018760
traf and tnf receptor associated protein	Dr.18071.2.A1_at	4.529896
Eukaryotic translation initiation factor 3, subunit E, b; eukaryotic translation initiation factor 3, subunit E, a	Dr.5119.1.A1_at	4.241705
Chemokine (C-X-C motif) ligand-12a (stromal cell-derived factor 1)	Dr.822.1.S3_at	3.862025
Cytochrome P-450, family-26, subfamily b, polypeptide 1	Dr.180.1.A1_at	3.092533
One-eyed pinhead	Dr.581.1.S1_at	2.272094
Similar to frizzled homolog 7b; frizzled homolog 7b; frizzled homolog 7a	Dr.5454.1.S1_at	2.128164
Axin-1	Dr.17733.1.S1_at	0.505078
Pre-B-cell leukemia transcription factor 1a; zgc:158824; pre-B-cell leukemia transcription factor 4; hypothetical LOC100004634; hypothetical protein LOC100150879	Dr.4926.1.S1_at	0.175405
Fin morphogenesis		
Activin A receptor, type I like	Dr.606.1.S2_at	11.01876
Chemokine (C-X-C motif) ligand 12a (stromal cell-derived factor 1)	Dr.822.1.S3_at	3.862025
Axin-1	Dr.17733.1.S1_at	0.505078
Golgi apparatus		
Stromal cell-derived factor-4	Dr.20092.1.S1_at	4.935459
Clathrin, light chain (Lca)	Dr.26380.1.A1_at	1.980670
UDP-Gal:betaGlcNAc beta 1,3-galactosyltransferase, polypeptide 2	Dr.11084.1.A1_at	1.701953
Partial optokinetic response-b	Dr.9437.1.A1_at	1.616283
Adaptor-related protein complex 1, sigma 1 subunit	Dr.18735.1.A1_at	0.409458
Beta-3-galactosyltransferase	DrAffx.1.31.S1_at	0.239189
Neuron differentiation		
T-box 24	Dr.18309.1.S1_at	5.279783
Acetylcholinesterase	Dr.15722.1.S1_at	4.445239
Neurogenin 1	Dr.555.1.S1_at	4.347208
Chemokine (C-X-C motif) ligand 12a (stromal cell-derived factor 1)	Dr.822.1.S3_at	3.862025
Semaphorin 3ab	Dr.8112.1.S1_at	3.372930
N-Ethylmaleimide-sensitive factor	Dr.9155.1.S1_at	0.541262
Survival of motor neuron protein interacting protein 1	Dr.2724.1.S1_at	0.480291
Pre-B-cell leukemia transcription factor 1a; zgc:158824; pre-B-cell leukemia transcription factor-4; hypothetical LOC100004634; hypothetical protein LOC100150879	Dr.4926.1.S1_at	0.175405
Organelle membrane		
Mitofusin-1	Dr.14642.1.S1_at	7.021206
Dullard homolog	Dr.7581.1.A1_at	5.367876
Translocase of outer mitochondrial membrane-40 homolog, like	Dr.15545.1.A1_at	4.868413
Cytochrome c oxidase subunit 1	Dr.20553.2.A1_at	2.223735
Clathrin, light chain (Lca)	Dr.26380.1.A1_at	1.980670
Translocase of inner mitochondrial membrane-17 homolog A (yeast)	Dr.3096.1.A1_at	1.925030
6.8 kDa mitochondrial proteolipid-like	Dr.1429.1.S1_at	1.575235
zgc:86898	Dr.661.1.S1_at	0.496350
Adaptor-related protein complex 1, sigma 1 subunit	Dr.18735.1.A1_at	0.409458
Carnitine palmitoyltransferase II	Dr.146.1.A1_at	0.308770
zgc:112986	Dr.20668.1.A1_at	0.307912
ADP-ribosylation factor-like 8Ba	Dr.7615.1.A1_at	0.249671
Asparagine-linked glycosylation 6 homolog (*S. cerevisiae*, alpha-1,3-glucosyltransferase)	Dr.9628.1.A1_at	0.199667

Fold change = (signal intensity of a gene in the muscle of crisp grass carp)/(signal intensity of the gene in the muscle of grass carp).
